# Comparison Study on the Estimation of the Spatial Distribution of Regional Soil Metal(loid)s Pollution Based on Kriging Interpolation and BP Neural Network

**DOI:** 10.3390/ijerph15010034

**Published:** 2017-12-26

**Authors:** Zhenyi Jia, Shenglu Zhou, Quanlong Su, Haomin Yi, Junxiao Wang

**Affiliations:** School of Geographic and Oceanographic Sciences, Nanjing University, Nanjing 210023, China; zhenyijay@smail.nju.edu.cn (Z.J.); njusuquanlong@163.com (Q.S.); 347806060@163.com (H.Y.); dz1427034@smail.nju.edu.cn (J.W.)

**Keywords:** soil metal(loid)s, spatial interpolation, BP neural network, cross validation

## Abstract

Soil pollution by metal(loid)s resulting from rapid economic development is a major concern. Accurately estimating the spatial distribution of soil metal(loid) pollution has great significance in preventing and controlling soil pollution. In this study, 126 topsoil samples were collected in Kunshan City and the geo-accumulation index was selected as a pollution index. We used Kriging interpolation and BP neural network methods to estimate the spatial distribution of arsenic (As) and cadmium (Cd) pollution in the study area. Additionally, we introduced a cross-validation method to measure the errors of the estimation results by the two interpolation methods and discussed the accuracy of the information contained in the estimation results. The conclusions are as follows: data distribution characteristics, spatial variability, and mean square errors (MSE) of the different methods showed large differences. Estimation results from BP neural network models have a higher accuracy, the MSE of As and Cd are 0.0661 and 0.1743, respectively. However, the interpolation results show significant skewed distribution, and spatial autocorrelation is strong. Using Kriging interpolation, the MSE of As and Cd are 0.0804 and 0.2983, respectively. The estimation results have poorer accuracy. Combining the two methods can improve the accuracy of the Kriging interpolation and more comprehensively represent the spatial distribution characteristics of metal(loid)s in regional soil. The study may provide a scientific basis and technical support for the regulation of soil metal(loid) pollution.

## 1. Introduction

Due to the continuously increasing intensities of industrial and agricultural activities, the soil environment is under enormous pressure. As a major aspect that affects soil environment quality, metal(loid) pollution has become increasingly prominent [1,2,3,4,5,6]. Spatial interpolation methods can obtain the spatial distribution of regional soil metal(loid) pollution, form a spatial sense of the degree of regional soil metal(loid) pollution, and refine assessment perspectives to specific point locations or local regions. Therefore, many researchers have studied the spatial distribution interpolation of soil metal(loid)s and other elements [7,8,9,10]. Kriging interpolation is the most widely used spatial interpolation method. It can provide a linear optimal unbiased estimation of the data at unknown sampling points in a region [11,12]. An artificial neural network (ANN) is a highly complex nonlinear dynamic system. It has very strong self-adapting, self-organizing, and self-learning capabilities [13,14,15]. It is especially suitable for multiple conditional, inaccurate, and ambiguous problems. Therefore, applying an artificial neural network to spatial estimations of soil metal(loid) pollution can produce scientific assessment results.

For studies on the accuracy of the spatial interpolation of soil metal(loid) pollution, some researchers have used a cross-validation method to verify the accuracy of interpolation. This method illustrates the accuracy of interpolation results very well [16,17,18]. However, apart from cross-validation, there are no other methods for error quantification. Spatial distribution estimation methods can further reveal more information about soil metal(loid) pollution in local regions, in addition to the basis of reflecting the overall degree of regional pollution. Juan and Lee used three nonparametric Kriging methods to delineate heavy metal contaminated soils [19]. Reza used the ordinary Kriging to estimate the spatial distribution of heavy metals in the soils of surrounding agricultural fields affected by mine drainage [20]. Hu et al. studied on spatial distribution of farmland soil heavy metals using BP neural network [21]. However, few researchers have conducted systematic summaries and comparisons of the assessment results by Kriging interpolation and BP neural network and synthetically combined the two assessment methods to comprehensively and accurately reveal regional metal(loid) pollution conditions. The few who did only touched on theoretical introductions, e.g., Zhang made a qualitative description about the difference between Kriging interpolation and BP neural network methods merely based on the maps of the spatial distribution [22,23,24]. This study lacked a quantitative discussion on the differences between Kriging interpolation and BP neural network assessment and the comprehensive assessment, and could not fully show the advantages and disadvantages of the two methods. Therefore, how to integrate Kriging interpolation and BP neural network methods to estimate the spatial distribution of the regional soil metal(loid) pollution, and to reveal the advantages and disadvantages between different methods by further analyzing the differences between the two methods systematically and quantitatively, has become an essential research topic in soil science.

Arsenic concentration (10.71 mg/kg) is lower than the soil background value (11.20 mg/kg) in Kunshan City, and the spatial variability is also relatively lower than other heavy metals (29.2%), such as Hg, Pb, Cd, Zn, Cu, etc. In contrast, cadmium content (0.22 mg/kg) is higher than the background value (0.10 mg/kg), and the spatial variability is the greatest among all soil metal(loid)s (88%), which seriously threatens the environment and human health [25,26]. Therefore, the selection of As and Cd is more representative to form a sharp contrast in the results of pollution assessment, which is conducive to the comparative study of the results of different estimation methods. The present work takes the following objectives: (1) to separately used Kriging interpolation and BP neural network interpolation methods to estimate the spatial distribution of the soil metal(loid)s pollution in Kunshan; (2) to combine the BP neural network densification and Kinging interpolation methods to comprehensively and accurately estimate the regional soil metal(loid) pollution conditions; (3) to compare the spatial distribution estimation results of different interpolation methods quantitatively, and further discuss their advantages and disadvantages systematically.

## 2. Materials and Methods

### 2.1. Sampling

**Figure 1:**
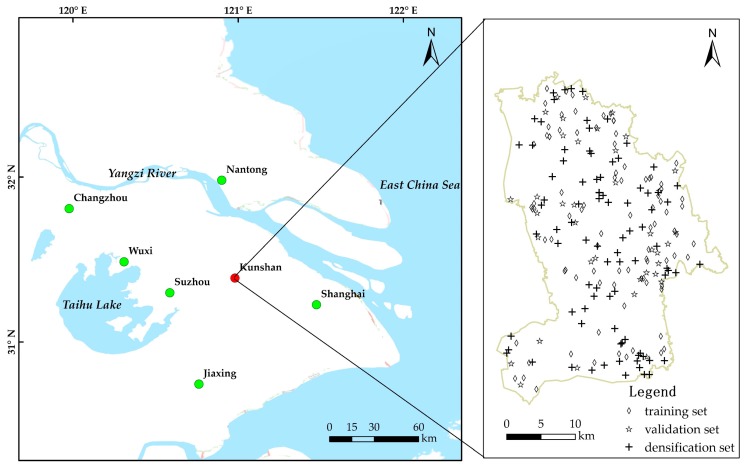
Study site and spatial distribution of each kind of sample. The 126 total samples were divided into two kinds of sets, including 88 training sets (70%) and 38 validation sets (30%), respectively. In addition, another 88 densification sets were added to increase the sampling density.

Kunshan City is in the southeast of Jiangsu Province, China, between Shanghai and Suzhou. It lies at 120°48′21″–121°09′04″ E and 31°06′34″–31°32′36″ N (Figure 1). Kunshan City has a typical northern subtropical monsoon climate. The mean annual temperature is 17.6 °C. The mean annual precipitation is 1200.4 mm. The soil of the city belongs to four soil types: Hydragric Anthrosols, Humic Gleysols, Gleyic Fluvisols, and Eutric Planosols. Hydragric Anthrosols were the main soil type and accounted for 93.8% of the soil in our study.

126 soil samples were collected according to the land use conditions and industrial and agriculture characteristics (Figure 1). The samples covered the major parent materials and soil types in the Kunshan City over five function zones that reflect urbanization, industrialization, and agricultural intensification processes and have high probabilities of potential soil pollution, including 26 chemical industrial zones; 18 dyeing, printing, and paper-making zones; 15 metallurgical and electroplating zones; 38 cultivation zones; and 29 vegetable fields. Except for the vegetable fields, where we sampled the soil in situ, we sampled the Hydragric Anthrosols approximately 50 m away from the factories in all other function zones. Surface soil samples (0–20 cm) were collected following a five-point mixing sampling method, and approximately 1.5 kg analysis samples were selected using the quartering method. Samples dried naturally, rocks and plant matter were eliminated. We ground it to pass through 100 mesh screen, and fully and homogenously mixed the material for later use.

This study used As and Cd as representative elements to study the difference in the estimation results of the degree of soil metal(loid) pollution under different methods. For Cd, we separately added concentrated hydrochloric acid and concentrated nitric acid, heated on hot plates with holes at 150 °C, added HF-HNO_3_-HClO_4_, heated on hot plates with holes at 200 °C to digest, and measured using the inductively coupled plasma mass spectrometry (ICP-MS, Perkin-Elmer SCIEX Inc., Elan 9000, Concord, ON, Canada) [27]. For As, we used 1:1 aqua regia and a boiling water bath to digest, followed by reduction-gasification-atomic fluorescence spectrometry (AFS, Jitian AFS-820, Beijing, China) to measure [28]. To control the analytical quality, 1 standard reference material sample was added into every 10 samples, and all samples were tested twice. The standard reference material of GSS-1 for soils, obtained from the Center of National Standard Reference Material of China. The results were consistent with the reference values, and the differences were all within 10%. Satisfactory recoveries were obtained for As (98%) and Cd (100%).

### 2.2. Geo-Accumulation Index

The geo-accumulation index is normally called the Muller index [29]. It can very accurately reflect the effect on heavy metal distribution of natural variation and human activity factors. It uses the background values of heavy metal concentrations in a study region as standards. It is an important pollution index for assessing regional heavy metal pollution. The equation is

(1) $$I_{geo}=\log_{2}[c_{i}/kB_{i}]$$

where *C_i_* is the measured concentration of soil heavy metal element *i* (mg·kg^−1^); *B_i_* is the regional background value of element *i* (mg·kg^−1^); and *k* is a correction coefficient, which is normally taken to be 1.5. Based on the geo-accumulation index *I_geo_*, we divided the degree of soil heavy metal pollution into five grades: zeroth grade when *I_geo_* ≤ 0, clean; first grade when 0 < *I_geo_* ≤ 1, slight pollution, second grade when 1 < *I_geo_* ≤ 2, intermediate pollution; third grade when 2 < *I_geo_* ≤ 3, heavy pollution; and fourth grade when *I_geo_* > 3, severe pollution.

### 2.3. Kriging

Kriging interpolation is also called a spatial local interpolation method. It can implement the linear optimal unbiased estimation of the data at unknown sampling points in a region. This method achieves linear interpolation through improving distance weights [30]. Software ArcGIS 10.3 (ESRI, Redlands, CA, USA) is used for Kriging interpolation. The equation is as follows: taking the variable *z*(*x*) over the object study region, where its value at point x∈A(i=1, 2…, n) is *z*(*x_i_*), the Kriging Interpolation result *z′*(*x*_0_) of the value *z*(*x*_0_) at *x*_0_ to be interpolated is the weighted sum of the values of the known sampling points *z*(*x_i_*) *(i* = 1, 2…, *n*), i.e.,

(2) $$z\prime (x_{0})=\sum_{i=1}^{n}\omega_{i}\cdot z(x_{i})$$

where *ω_i_* is a weight coefficient to be determined. Using the unbiased and least square errors as constraints, we can obtain the equations to solve the weight coefficient to be determined, *ω_i_*, as

(3) $$\sum_{i=1}^{n}\omega_{1}C(x_{i},y_{j})+\mu =C_{0}(x_{i},y_{j})\left(j=1,2\ldots ,n\right)$$

(4) $$\sum_{i=1}^{n}\omega_{i}=1$$

### 2.4. BP Neural Network

An artificial neural network (ANN), simplified as neural network, is a data processing model constructed under the inspiration of biological neural networks [31]. A neural network conducts calculations using a large amount of interconnecting artificial neurons. It changes its structure based on external information. Researchers perform input data modeling by adjusting the weights and thresholds among neurons, thereby ultimately achieving the capability to solve real problems. The BP neural network is a type of forward neural network. The characteristics of this type of network are that, during the process of calculating output values, the input values sequentially propagate from the direction input layer—hidden layer—output layer, finally yielding the output. This process is opposite from feedback neural networks. The transfer function of a BP neural network can involve nonlinear functions [32]. The most common functions include logsig, tansig, and linear purelin functions. The former two are sigmoid functions. If the last layer of a BP neural network contains sigmoid neurons, the network output values are limited within a small range with absolute values less than 1. If it contains linear neurons, the whole network output can have any value. The BP network structure is multi-layered, i.e., in addition to the input layer and output layer, it has several hidden layers. It has the capability to process linear inseparable problems. In real applications, researchers normally set up one hidden layer. The number of input neurons is *R*. The number of hidden layers is *S*^1^. The number of neurons in the output layer is *S*^2^. The transfer functions of the hidden layer and output layer are logsig and purelin functions, respectively. This network will ultimately output a vector with *S*^2^ nodes. We use *ν_i_* to represent the output of the *i*th neuron in the output layer and *n* as the number of iterations. The actual network output is *Y*(*n*) = [*v*_1_, *v*_2_, …*v_s_*^2^]. If the expected network output is *d*(*n*) = [*d*_1_, *d*_2_, …*d_s_*^2^], then the error signal of the *n*th iteration is defined as *e_i_*(*n*) = *d_i_*(*n*) – *Y_i_*(*n*), and the error energy is defined as

(5) $$e(n)=\frac{1}{2}\sum_{i=1}^{s^{2}}e_{i}^{2}(n)$$

BP network learning belongs to the error minimization type of supervised learning, i.e., it requires a learning sample set with known output and minimizes the error between the expected output and actual output through training. The establishment and training of BP neural networks are implemented using the neural network toolbox in software MATLAB R2014a (MathWorks Inc., Natick, MA, USA).

### 2.5. Cross-Validation

This study used a cross-validation method to measure the errors of the estimation results by different interpolation methods. We used the mean square error (MSE) index for evaluation. Based on this method, the sample data are divided into a 70% training set and 30% validation set. Normally, data groups are randomly selected. However, as the 70% training set after division can affect the original layout configuration to some extent, we divided the data so that the 70% training set represented the original distribution characteristics. This study used a random selection method for data division and obtained 88 training sets and 38 validation sets. We used the geo-accumulation index method to find the geo-accumulation index of the As and Cd concentrations and used Kriging interpolation for qualitative and quantitative analysis and comparison of the spatial distribution of the pollution. Figure 1 shows the spatial distribution map of the training set and validation set.

### 2.6. Interpolation Process

The training set includes 88 sample data in total. Kriging interpolation requires that the data satisfy a normal distribution. Therefore, we first need to test the normality of the data. Before the normality test, we first identified and eliminated anomalous data. We used the Grubbs test method for statistical analysis of the training data set. The As data do not contain anomalous values. The Cd data contain four anomalies. Combined with the actual situations of the sample locations, the Cd concentrations at the four locations are indeed too large: they are outliers, and hence, we eliminated them. We then used software SPSS 20.0 (IBM SPSS Inc., Chicago, IL, USA) to conduct the K–S test on the geo-accumulation indices of As and Cd. The sig values are 0.906 and 0.13, respectively. The data follow a normal distribution at the 0.05 significance level.

### 2.7. BP Neural Network Simulation and Densification

During actual field sampling work, many factors limited the soil heavy metal(loid) samples to be analyzed. Take the data in this study as an example. We only had 126 total samples based on function zones. After dividing the samples into a training set and validation set, only 88 samples were used in modeling. The other 38 samples were needed for validation as measured data. This necessity further decreased the amount of information in the analysis data and affected the reliability of the experimental results. Accordingly, we introduced the training sample data to the neural network to learn and used the successfully learned network to appropriately increase the sampling location density. We then incorporated Kriging interpolation to further estimate the spatial distribution of soil metal(loid) pollution. We also introduced an elevation factor during the network training process so that the estimation results more accurately reflect the reality of soil metal(loid) pollution in the study region. The experimental data related to the BP network are shown in Table 1.

After BP neural network training, we could conduct spatial simulation (interpolation) on the As and Cd geo-accumulation indices at unknown spatial sample locations. We input some spatial information (longitude, latitude, and elevation) from the validation set of the sample data into the network to simulate and implemented spatial estimation of the soil metal(loid) geo-accumulation index by the BP neural network. We then compared the measured data of the validation set to achieve quantitative error analysis of the spatial interpolation by the BP neural network.

## 3. Results

### 3.1. The Spatial Distribution Estimation by Kriging Interpolation and BP Neural Network

The mean square errors of the spatial estimation results by Kriging interpolation for As and Cd are 0.0804 and 0.2983, respectively (details are in the Supplementary Materials Table S1). The mean square errors of the simulation results by BP neural network for As and Cd are 0.0661 and 0.1743, respectively. Based on the overall effect of the BP neural network interpolation for the 38 validation set data, the estimation is more accurate than the Kriging interpolation. However, the errors in the simulation of some local points may be large. Empirical analysis indicates that even if the estimation by this method is more accurate than Kriging interpolation, it is difficult to implement the simulation of the whole region using the BP neural network model alone, and we cannot analyze and discuss the spatial distribution of the estimation results. Therefore, we sought to combine Kriging interpolation and the BP neural network to estimate the spatial distribution of regional soil heavy metal(loid) pollution. On one hand, this approach implements the spatial distribution study of the estimation results through the BP neural network. One the other hand, it also improves the accuracy of the Kriging interpolation. Especially in situations with a small number of samples, using the BP network to increase data density can produce estimation results with a more accurate spatial distribution and more abundant information.

We randomly selected 88 locations to increase the sampling density in the study region (Figure 1). After retrieving the coordinates and elevation information of the densification locations, we can input the data into the previously established BP network for data simulation. Finally, we obtained 176 training and densification data points. We then conducted Kriging interpolation on the data to further examine the variation of the estimation results for the regional soil metal(loid)s pollution by Kriging interpolation after increasing the data density.

### 3.2. Spatial Distribution Comparison

**Figure 2:**
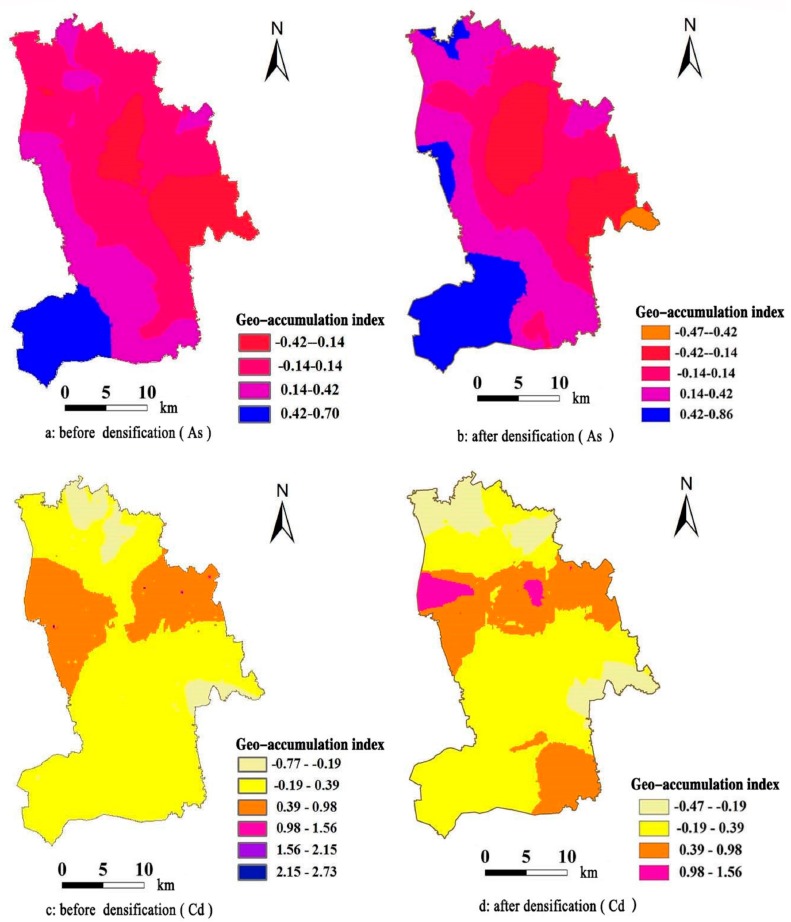
Spatial distribution maps of the geo-accumulation indices of As and Cd before and after densification. The classification of the geo-accumulation index was obtained by using natural break interval approach. (**a**) The geo-accumulation index of As before densification; (**b**) the geo-accumulation index of As after densification; (**c**) the geo-accumulation index of Cd before densification; (**d**) the geo-accumulation index of Cd after densification.

Figure 2 is the spatial distribution maps of the geo-accumulation indices of As and Cd before and after densification. Based on the Figure 2, the spatial distribution configuration of the geo-accumulation indices of As and Cd before and after densification does not change much. However, the spatial distribution of the geo-accumulation indices after densification contains more abundant information. Based on the interval ranges of the indices, the interval range of the As pollution index increases, and the interval range of the Cd pollution index decreases.

**Figure 3:**
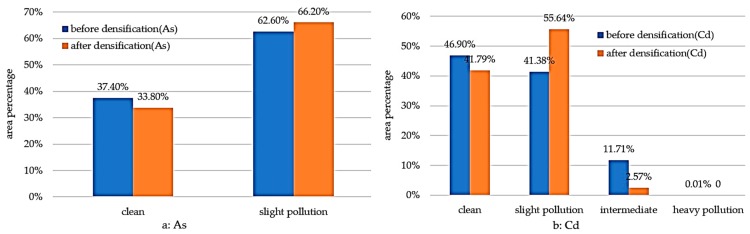
Statistics of each pollution level before and after densification. For As, the pollution grades were clean and slight pollution. For Cd, the pollution grades were clean, slight, intermediate, and heavy pollution. (**a**) The area percentage of each pollution level before and after densification of As, respectively; (**b**) the area percentage of each pollution level before and after densification of Cd, respectively.

We then performed statistical analysis on the regional mean geo-accumulation indices after interpolation. The As values are 0.0955 and 0.1458 before and after densification, respectively. The Cd values are 0.1364 and 0.1934 before and after densification, respectively. The assessment results of the overall regional pollution degree of the two elements after densification both increase. Finally, we performed statistical analysis on the area percentages of each pollution grade and obtained the statistical histogram (Figure 3). For As, the pollution grades are clean and slight pollution. After densification, the area percentage of slight pollution increases. Therefore, the overall pollution degree increases. For Cd, the pollution grades are clean, slight, intermediate, and heavy pollution. However, heavy pollution only constitutes 0.01% before densification and can be basically neglected. After densification, the area percentage of slight pollution increases by approximately 14%, while the area percentages of clean and intermediate pollution both decrease by different degrees. In particular, intermediate pollution decreases by a large amount, from 11.71% to only 2.57%. Based on the overall variation conditions, the variability of the spatial distribution of Cd pollution decreases, showing a centralized trend.

### 3.3. The Spatial Distribution Estimation Results before and after Densification

**Figure 4:**
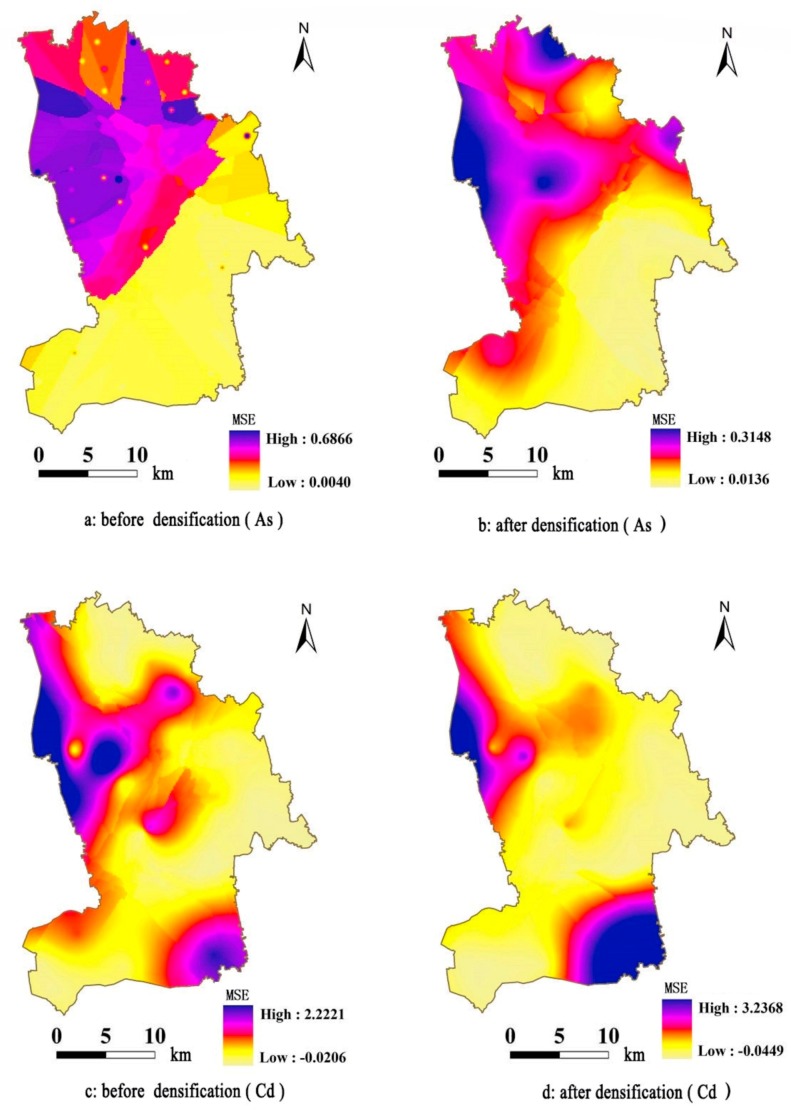
Spatial distribution maps of the mean square errors of the estimation results before and after densification. (**a**) The MSE of the geo-accumulation indices of As before densification; (**b**) the MSE of the geo-accumulation indices of As after densification; (**c**) the MSE of the geo-accumulation indices of Cd before densification; (**d**) the MSE of the geo-accumulation indices of Cd after densification.

After obtaining the spatial distribution map of the Kriging interpolation of the study region, we can extract the soil metal(loid) geo-accumulation indices at the sampling locations of the validation set. After extracting the estimation values of the geo-accumulation indices before and after densification and quantitatively analyzing the statistics of the errors, the mean square errors of the geo-accumulation indices of As and Cd after densification are 0.0594 and 0.2177, respectively (detailed in the Supplementary Materials Table S2). They decrease by different degrees compared to the mean square errors before densification, indicating that the accuracy of the Kriging interpolation results increase after using the BP neural network to increase the density of the sample data. Combining the two interpolation methods can combine the advantages of interpolation accuracy and visualization. In addition, we can further conduct spatial Kriging interpolation on the square errors of the validation set locations to compare the spatial distribution characteristics of the square errors of the estimation results before and after densification. The results are shown in Figure 4.

In Figure 4a,b are the mean square error values of the geo-accumulation indices of As before and after densification, respectively. Based on the interval range of the error, the maximum MSE after densification is less than half the value before densification. The interval range decreases by a large magnitude. In the spatial distribution, the spatial variation of the MSE after densification is much smoother. The overall distribution configurations are similar. Figure 4c,d are the square errors of the geo-accumulation indices of Cd before and after densification, respectively. Based on the interval range of the errors, the maximum MSE after densification is 3.2368, larger than before densification. The interval range increases. On the spatial distribution, the high error values after densification decrease to a certain degree in the northwest of the region. However, the error further concentrates in the fan region in the southeast of the region, and the area increases significantly. The error configurations in the other regions are similar.

Summarizing the above analysis results, we used the BP neural network and Kriging interpolation to estimate the spatial distribution of the soil metal(loid) geo-accumulation indices. This method is better than the simple Kriging method in terms of estimation result accuracy. Meanwhile, it makes up for the discrepancy that it is difficult to implement spatial visualization analysis using the BP neural network, which has good practical application significance.

## 4. Discussion

We used a BP neural network to conduct simulation interpolation for the soil metal(loid) geo-accumulation indices. Empirical analysis indicates that even if the estimation by this method is more accurate than Kriging interpolation, it is difficult to implement the simulation of the whole region using the BP neural network model alone, and we cannot analyze or discuss the spatial distribution of the estimation results. Some researchers combined the BP neural network and Kriging interpolation to study the spatial distribution of soil metal(loid) pollution with a small number of samples and obtained good study results. However, these studies lacked quantitative analysis on the error of the simulation results by the BP neural network. Therefore, we combined Kriging interpolation and the BP neural network to estimate the spatial distribution of regional soil metal(loid) pollution. This approach implements the spatial distribution study of the estimation results by the BP neural network and also improves the accuracy of Kriging interpolation. Especially in situations with a small number of samples, using the BP network to increase data density can produce estimation results with a more accurate spatial distribution and more abundant information.

However, the Kriging interpolation method can have a strong smoothing effect on the results, such as decreasing extreme values and decreasing square error [12]. Spatial interpolation results have uniqueness and do not represent the random variation characteristics of the spatial distribution of soil metal(loid) pollution in the study region. To represent the random variation characteristics of the spatial distribution of soil metal(loid) pollution in the study region, we discuss the use of sequential Gaussian simulation for spatial interpolation estimation, to more comprehensively reflect the reality of the soil metal(loid) pollution in the study region. Sequential Gaussian simulation is a typical type of conditional simulation. It is suitable to quantitatively depict the heterogeneity and uncertainty of one property of the study region [33]. It integrates different information through random modeling of the property values and includes the uncertainty and correlation of the information in the model. It emphasizes the effect of probability models on the results. Sequential Gaussian simulation results are provided in the Supplementary Materials Table S3.

We analyzed the descriptive statistical characteristics of the results using the Kriging method, sequential Gaussian simulation, and BP neural network densification interpolation, including the maximum, minimum, mean, standard deviation, kurtosis, and skewness. They are shown in Table 2.

Based on the interval ranges of the values, while the interval lengths of the Kriging interpolation result and sequential Gaussian simulation of Cd are close, the interval length of sequential Gaussian simulation is the largest, reflecting that the randomness of its estimation result is strong. For the mean values, the sequential Gaussian simulation is the smallest. The mean of the results of BP neural network densification interpolation is the largest. For the standard deviations, the standard deviation of the sequential Gaussian simulation is the largest, which is related to the randomness of the estimation result. The kurtosis values of the three do not show any significant difference. They are all steeper than the standard normal distribution. For skewness, the result by sequential Gaussian simulation is close to 0, indicating that its distribution pattern is very similar to normal distribution. While Kriging interpolation and BP network densification interpolation results all show large positive values, i.e., they show clear right-skewed distributions.

We introduced the variogram theory in geostatistics to further analyze the spatial variation characteristics of the estimation results. The semivariogram requires that the sample data follow a normal distribution or follow a normal distribution after transformation [34]. Because the results of the Kriging interpolation and BP network densification interpolation do not follow a normal distribution, we conducted a logarithmic normal transformation and passed a significance test. We used different variogram models to fit the estimation results by different methods and the original sample data and then selected the fitting model with the largest coefficient of determination R^2^ and the smallest residual sum of squares (RSS). We finally obtained the following Table 3 of the relevant parameters of the semivariogram functions of metal(loid)s.

From the coefficient of determination R^2^, the coefficients of determination of the fitting models all reach the *p* = 0.01 significance level. The maximum RSS is also approximately 0.01, indicating that the selected variograms can accurately fit the data estimation results by these methods. The variograms of the Kriging interpolation and BP network densification interpolation are both Gaussian models, while the original data and sequential Gaussian simulation interpolation are spherical or index models. For the block gold values and abutment values, the values of the sequential Gaussian simulation and original data are close and are significantly higher than the interpolation results by the other two methods, indicating that the sequential Gaussian simulation results reproduced the spatial variation characteristics of the original data very well. The block gold value/abutment value is also called the block gold coefficient, indicating the magnitude of the variation feature of the samples. The block gold coefficients of the Kriging interpolation and BP network densification interpolation are less than 5%, which also indicates that the spatial random variability of the sequential Gaussian simulation result and the original data are significantly stronger than the Kriging interpolation and BP network densification interpolation. The result by sequential Gaussian simulation maintains the spatial variability of the original data well, while the results of the Kriging interpolation and BP network intensification interpolation cause strong spatial autocorrelation and cannot reproduce the spatial variability of the original data. Therefore, considering the spatial variation of the results, the result of the sequential Gaussian simulation is significantly better than the other two.

We used a cross-validation method to quantitatively compare the errors of the results by the four estimation methods, as shown in Table 4.

From the Table 4, the MSE of the sequential Gaussian simulation is the largest, i.e., the accuracy of the estimation result is the lowest. This result is caused by the uncertainty estimation of this method. From the aspect of practical application, applying a single sequential Gaussian simulation result to regional soil metal(loid) pollution assessment is not very meaningful. This problem is also not the application focus of sequential Gaussian simulation. This simulation method focuses on the comprehensiveness of the information contained in the estimation results for the soil metal(loid) concentration. During simulation, it considers the effects of many random factors including sampling method, depth, and capacity. It uses probability as the theoretical basis and considers the simulation at specific locations in the study region as implementations of certain probabilities. Therefore, using sequential Gaussian simulation multiple times can produce a group of different estimation values at specific locations. It can provide a more comprehensive understanding of the information on the degree of soil metal(loid) pollution at specific locations. It can also provide scientific and effective evidence for preventing soil metal(loid) pollution in some regions. The mean square errors of the BP neural network and Kriging interpolation after BP neural network densification decrease by different degrees compared to the simple Kriging interpolation, indicating that the accuracy of the estimation result by the BP neural network is relatively high. Meanwhile, the accuracy of the estimation result by Kriging interpolation after increasing the data density also increases. The fundamental reasons for the improved accuracy are the strong mapping capability of the BP neural network and considering the sampling location elevation in the spatial estimation. Therefore, we can further consider the BP neural network simulation from the perspective of the accuracy of the spatial estimation results. To consider the objective of spatial visualization analysis, the effect would be better if we combined the BP neural network simulation with Kriging interpolation for spatial estimation.

## 5. Conclusions

In this study, we conclude that the accuracy of the estimation result from BP neural work is the highest. The accuracy of the estimation result using Kriging interpolation is lower. The spatial distribution of the geo-accumulation indices after densification contains more abundant information than its original. It can better reflect the local soil metal(loid) pollution conditions. It indicates that the larger the sampling density, the smaller the error of interpolation result [35]. As a result, combing the two methods can implement the spatial distribution study on the estimation results of the BP neural network, and improve the accuracy of the Kriging interpolation method. The method of combing BP neural network and Kriging interpolation is beneficial to precision management and remediation of regional soil metal(loid) pollution, which will help reduce human health risks.

We introduced an elevation factor during the BP neural network training process so that the estimation results more accurately reflect the reality of soil metal(loid) pollution in the study region. As we know, soil metal(loid) concentration is not only affected by the topography, but also closely related to other factors such as soil properties, geological background and land use, etc. [36,37]. Therefore, in the future, we will do more work to verify whether the introduction of multiple correlation factors in the BP neural network training can further improve the interpolation accuracy.

## Figures and Tables

**Table 1:** Relevant test parameters of the BP neural network.

Element	Training Function	Hidden Layer Neuron Number	Training Termination Error	Training Termination Gradient	Iteration	Validation Check
As	traingd	12	0.02	0.0186	614	pass
Cd	traingd	12	0.02	0.0131	880	pass

**Table 2:** Descriptive statistical analysis results of the As and Cd geo-accumulation indices.

Element	Interpolation Method	Minimum	Maximum	Mean	Standard Deviation	Kurtosis	Skewness
As	Kriging	−0.3243	0.6987	0.0975	0.2312	2.7519	0.6181
	Sequential Gaussian	−1.0399	1.1781	0.0697	0.3351	2.8923	0.0297
	BP network	−0.4687	0.805	0.151	0.3003	2.392	0.2575
Cd	Kriging	−0.7749	2.7329	0.1439	0.2994	3.0325	0.9433
	Sequential Gaussian	−1.4776	1.9031	0.113	0.4642	2.9687	0.1009
	BP network	−0.4663	1.1883	0.1966	0.3826	2.4198	0.6704

**Table 3:** Semivariogram model and parameters of the estimation results by different interpolation methods.

Element	Interpolation (Original Data)	Variogram Model	Block Gold Value	Abutment Value	Block Gold Value/Abut-Ment Value	Range	Coefficient of Determination R^2^	Residual Sum of Squares
As	Original data	Spherical	0.0555	0.1400	39.64	31,450.00	0.455	0.0130
	Kriging interpolation	Gaussian	0.0001	0.0892	0.11	28,422.95	0.920	0.0015
	Sequential Gaussian simulation	Spherical	0.0582	0.1174	49.57	18,540.00	0.602	0.0025
	BP network densification interpolation	Gaussian	0.0028	0.1116	2.51	20,594.08	0.541	0.0195
Cd	Original data	Spherical	0.1192	0.2394	49.79	17,900.00	0.645	0.0113
	Kriging interpolation	Gaussian	0.0043	0.1126	3.82	14,098.89	0.992	0.0002
	Sequential Gaussian simulation	Index	0.1046	0.2162	48.38	8400.00	0.871	0.0013
	BP network densification interpolation	Gaussian	0.0016	0.1722	0.93	11,847.23	0.929	0.0046

Note: R_0.05_ = 0.336 (R^2^ = 0.113), R_0.01_ = 0.410 (R^2^ = 0.168).

**Table 4:** Comparison of the mean square errors of the results by each estimation method.

Element	BP Network Densification Interpolation	BP Network	Sequential Gaussian Simulation	Kriging Interpolation
As	0.0594	0.0661	0.1482	0.0804
Cd	0.2177	0.1743	0.3246	0.2983

## Supplementary Materials

The following are available online at www.mdpi.com/1660-4601/15/1/34/s1, Table S1: The mean square errors of the geo-accumulation index by Kriging interpolation and BP network for As and Cd, Table S2: The mean square errors of the geo-accumulation index of As and Cd before and after densification for As and Cd, Table S3: The mean square errors of the geo-accumulation index by the sequential Gaussian simulation.
