# Supplementary material for: Comparison Study on the Estimation of the Spatial Distribution of Regional Soil Metal(loid)s Pollution Based on Kriging Interpolation and BP Neural Network
Source: Int J Environ Res Public Health. 2017 Dec 26;15(1):34. doi: 10.3390/ijerph15010034 (PMC5800134; doi:10.3390/ijerph15010034)
Supplement: Supplementary file 1 [file ijerph-15-00034-s001.docx]

**Title: Comparison study on the estimation of the spatial distribution of regional soil heavy metal pollution based on Kriging interpolation and BP neural network**

Zhenyi Jia, Shenglu Zhou, Quanlong Su, Haomin Yi, Junxiao Wang

**Corresponding author:** Shenglu Zhou

**Affiliations:**

School of Geographic and Oceanographic Sciences, Nanjing University, 163 Xianlin Road, Nanjing, 210023, P.R. China

**Table S1** The mean square errors of the geo-accumulation index by Kriging interpolation and BP network for As and Cd

| Sample NO. | The geo-accumulation index of As | | | | | The geo-accumulation index of Cd | | | | |
| --- | --- | --- | --- | --- | --- | --- | --- | --- | --- | --- |
|  | Measured value | Kriging interpolation | MSE | BP network | MSE | Measured value | Kriging interpolation | MSE | BP network | MSE |
| 1 | 0.4857 | 0.6090 | 0.0152 | 0.8343 | 0.1215 | -0.1184 | -0.0660 | 0.0027 | -0.2391 | 0.0146 |
| 2 | 0.4517 | 0.4768 | 0.0006 | 0.8973 | 0.1986 | 0.2630 | -0.0001 | 0.0692 | 0.0517 | 0.0446 |
| 3 | 0.6664 | 0.6092 | 0.0033 | 0.9295 | 0.0692 | -0.2065 | -0.0844 | 0.0149 | -0.2932 | 0.0075 |
| 4 | 0.2135 | 0.1573 | 0.0032 | 0.6136 | 0.1601 | -0.9913 | -0.0387 | 0.9075 | -0.9822 | 0.0001 |
| 5 | 0.2771 | 0.5612 | 0.0807 | 0.4980 | 0.0488 | -0.7370 | -0.0844 | 0.4259 | -0.1685 | 0.3232 |
| 6 | 0.1460 | 0.0098 | 0.0185 | 0.1266 | 0.0004 | -0.0265 | -0.0675 | 0.0017 | 0.1767 | 0.0413 |
| 7 | -0.3934 | -0.2220 | 0.0294 | -0.1661 | 0.0517 | -0.0995 | -0.2366 | 0.0188 | -0.1723 | 0.0053 |
| 8 | -0.1546 | -0.1116 | 0.0018 | -0.0493 | 0.0111 | -0.0176 | -0.0146 | 0,0000 | 0.0017 | 0.0004 |
| 9 | -0.4860 | -0.1868 | 0.0895 | -0.1083 | 0.1427 | -0.4356 | -0.2366 | 0.0396 | -0.1255 | 0.0962 |
| 10 | -0.1559 | -0.1572 | 0.0000 | -0.0906 | 0.0043 | -0.2578 | -0.1532 | 0.0109 | -0.0843 | 0.0301 |
| 11 | 0.0100 | -0.1858 | 0.0383 | -0.1473 | 0.0247 | -0.3663 | -0.2366 | 0.0168 | -0.1507 | 0.0465 |
| 12 | -0.2747 | 0.0529 | 0.1073 | -0.0120 | 0.0690 | -0.1864 | -0.0563 | 0.0169 | 0.2191 | 0.1644 |
| 13 | -0.0570 | 0.0297 | 0.0075 | 0.0189 | 0.0058 | 0.7839 | -0.0563 | 0.7059 | 0.0105 | 0.5981 |
| 14 | -0.2890 | -0.2004 | 0.0079 | -0.1506 | 0.0192 | 0.1572 | -0.0791 | 0.0559 | -0.1406 | 0.0887 |
| 15 | -0.0595 | -0.2030 | 0.0206 | -0.0045 | 0.0030 | -0.2065 | -0.0046 | 0.0407 | -0.1410 | 0.0043 |
| 16 | -0.1428 | -0.2025 | 0.0036 | -0.2213 | 0.0062 | -0.1964 | -0.0500 | 0.0214 | -0.0983 | 0.0096 |
| 17 | 0.0297 | 0.2563 | 0.0513 | 0.3304 | 0.0904 | -0.4475 | 0.5613 | 1.0177 | -0.0868 | 0.1301 |
| 18 | -0.4152 | -0.2053 | 0.0441 | -0.0666 | 0.1215 | -0.3551 | 0.1426 | 0.2477 | -0.0930 | 0.0687 |
| 19 | -0.2977 | -0.2096 | 0.0078 | -0.2450 | 0.0028 | 0.1175 | 0.1749 | 0.0033 | 0.0494 | 0.0046 |
| 20 | -0.1823 | -0.1014 | 0.0066 | -0.3890 | 0.0427 | 0.1651 | 0.5581 | 0.1545 | -0.1292 | 0.0866 |
| 21 | -0.0376 | 0.3142 | 0.1237 | 0.6807 | 0.5159 | -0.2578 | 0.5744 | 0.6925 | 0.3571 | 0.3781 |
| 22 | -1.0136 | -0.1078 | 0.8205 | -0.7978 | 0.0466 | -0.3551 | 0.5581 | 0.8339 | -0.2698 | 0.0073 |
| 23 | -0.0339 | 0.0093 | 0.0019 | 0.0422 | 0.0058 | -0.6939 | 0.5581 | 1.5675 | -0.2295 | 0.2157 |
| 24 | -0.2410 | 0.2733 | 0.2645 | -0.3372 | 0.0093 | -0.8428 | 0.6505 | 2.2297 | 0.0869 | 0.8643 |
| 25 | 0.5761 | 0.2336 | 0.1173 | 0.9043 | 0.1077 | 0.2384 | 0.5986 | 0.1298 | 0.1177 | 0.0146 |
| 26 | 0.1267 | 0.0447 | 0.0067 | 0.2191 | 0.0085 | 0.5590 | 0.7680 | 0.0437 | 0.5726 | 0.0002 |
| 27 | 0.1713 | -0.1590 | 0.1091 | 0.0680 | 0.0107 | 0.1611 | 0.5971 | 0.1901 | -0.1212 | 0.0797 |
| 28 | -0.3950 | 0.1512 | 0.2983 | -0.4290 | 0.0012 | 0.2931 | 0.7680 | 0.2255 | 1.6358 | 1.8028 |
| 29 | -0.3388 | -0.1727 | 0.0276 | -0.4002 | 0.0038 | -0.6384 | 0.2675 | 0.8207 | -0.2091 | 0.1843 |
| 30 | -0.3064 | 0.1363 | 0.1960 | -0.0403 | 0.0708 | -0.0902 | -0.2026 | 0.0126 | -0.2762 | 0.0346 |
| 31 | -0.0196 | -0.0421 | 0.0005 | 0.1421 | 0.0261 | -0.2065 | 0.2675 | 0.2247 | -0.1116 | 0.0090 |
| 32 | 0.1006 | 0.1658 | 0.0043 | -0.1321 | 0.0541 | -0.3002 | -0.0539 | 0.0607 | 0.3779 | 0.4598 |
| 33 | 0.1555 | -0.0630 | 0.0478 | -0.0868 | 0.0587 | -0.7370 | -0.1891 | 0.3002 | -0.5764 | 0.0258 |
| 34 | -0.2648 | 0.1002 | 0.1332 | 0.2349 | 0.2497 | -0.1375 | -0.1507 | 0.0002 | -0.1369 | 0.0000 |
| 35 | 0.1941 | 0.0203 | 0.0302 | 0.2912 | 0.0094 | -0.1279 | -0.1565 | 0.0008 | -0.3979 | 0.0729 |
| 36 | 0.0401 | 0.1143 | 0.0055 | 0.2813 | 0.0582 | -0.5590 | -0.1196 | 0.1930 | -0.8650 | 0.0936 |
| 37 | -0.4893 | 0.0808 | 0.3250 | -0.5830 | 0.0088 | -0.2895 | -0.1799 | 0.0120 | 0.4069 | 0.4850 |
| 38 | 0.2125 | 0.1439 | 0.0047 | 0.4822 | 0.0727 | -0.0444 | -0.2042 | 0.0256 | -0.4048 | 0.1299 |
| **Mean** | **-0.0577** | **0.0532** | **0.0804** | **0.0826** | **0.0661** | **-0.1892** | **0.1138** | **0.2983** | **-0.0642** | **0.1743** |

**Table S2** The mean square errors of the geo-accumulation index of As and Cd before and after densification for As and Cd

| Sample NO. | The geo-accumulation index of As | | | | | The geo-accumulation index of Cd | | | | |
| --- | --- | --- | --- | --- | --- | --- | --- | --- | --- | --- |
|  | Measured value | before  densification | MSE | after densification | MSE | Measured value | before  densification | MSE | after densification | MSE |
| 1 | 0.4857 | 0.6090 | 0.0152 | 0.6666 | 0.0327 | -0.1184 | -0.0660 | 0.0027 | -0.1486 | 0.0009 |
| 2 | 0.4517 | 0.4768 | 0.0006 | 0.6163 | 0.0271 | 0.2630 | -0.0001 | 0.0692 | 0.0544 | 0.0435 |
| 3 | 0.6664 | 0.6092 | 0.0033 | 0.7276 | 0.0037 | -0.2065 | -0.0844 | 0.0149 | -0.1518 | 0.0030 |
| 4 | 0.2135 | 0.1573 | 0.0032 | 0.2986 | 0.0072 | -0.9913 | -0.0387 | 0.9075 | -0.7721 | 0.0480 |
| 5 | 0.2771 | 0.5612 | 0.0807 | 0.7428 | 0.2169 | -0.7370 | -0.0844 | 0.4259 | -0.1537 | 0.3402 |
| 6 | 0.1460 | 0.0098 | 0.0185 | 0.0191 | 0.0161 | -0.0265 | -0.0675 | 0.0017 | -0.0021 | 0.0006 |
| 7 | -0.3934 | -0.2220 | 0.0294 | -0.2272 | 0.0276 | -0.0995 | -0.2366 | 0.0188 | -0.2679 | 0.0284 |
| 8 | -0.1546 | -0.1116 | 0.0018 | -0.1273 | 0.0007 | -0.0176 | -0.0146 | 0,0000 | -0.1716 | 0.0237 |
| 9 | -0.4860 | -0.1868 | 0.0895 | -0.1886 | 0.0884 | -0.4356 | -0.2366 | 0.0396 | -0.2679 | 0.0281 |
| 10 | -0.1559 | -0.1572 | 0.0000 | -0.1440 | 0.0001 | -0.2578 | -0.1532 | 0.0109 | -0.2135 | 0.0020 |
| 11 | 0.0100 | -0.1858 | 0.0383 | -0.1637 | 0.0302 | -0.3663 | -0.2366 | 0.0168 | -0.1897 | 0.0312 |
| 12 | -0.2747 | 0.0529 | 0.1073 | 0.0519 | 0.1067 | -0.1864 | -0.0563 | 0.0169 | 0.0354 | 0.0492 |
| 13 | -0.0570 | 0.0297 | 0.0075 | -0.0094 | 0.0023 | 0.7839 | -0.0563 | 0.7059 | 0.0012 | 0.6126 |
| 14 | -0.2890 | -0.2004 | 0.0079 | -0.1753 | 0.0129 | 0.1572 | -0.0791 | 0.0559 | -0.0773 | 0.0550 |
| 15 | -0.0595 | -0.2030 | 0.0206 | -0.1748 | 0.0133 | -0.2065 | -0.0046 | 0.0407 | -0.0703 | 0.0185 |
| 16 | -0.1428 | -0.2025 | 0.0036 | -0.1900 | 0.0022 | -0.1964 | -0.0500 | 0.0214 | -0.1367 | 0.0036 |
| 17 | 0.0297 | 0.2563 | 0.0513 | 0.4466 | 0.1738 | -0.4475 | 0.5613 | 1.0177 | 0.5286 | 0.9528 |
| 18 | -0.4152 | -0.2053 | 0.0441 | -0.1632 | 0.0635 | -0.3551 | 0.1426 | 0.2477 | 0.07 | 0.1807 |
| 19 | -0.2977 | -0.2096 | 0.0078 | -0.189 | 0.0118 | 0.1175 | 0.1749 | 0.0033 | 0.1115 | 0.0000 |
| 20 | -0.1823 | -0.1014 | 0.0066 | -0.2016 | 0.0004 | 0.1651 | 0.5581 | 0.1545 | -0.1037 | 0.0722 |
| 21 | -0.0376 | 0.3142 | 0.1237 | 0.4180 | 0.2075 | -0.2578 | 0.5744 | 0.6925 | 0.8815 | 1.2980 |
| 22 | -1.0136 | -0.1078 | 0.8205 | -0.8821 | 0.0173 | -0.3551 | 0.5581 | 0.8339 | 0.3952 | 0.5629 |
| 23 | -0.0339 | 0.0093 | 0.0019 | -0.0079 | 0.0007 | -0.6939 | 0.5581 | 1.5675 | 0.5088 | 1.4465 |
| 24 | -0.2410 | 0.2733 | 0.2645 | -0.2752 | 0.0012 | -0.8428 | 0.6505 | 2.2297 | -0.9592 | 0.0136 |
| 25 | 0.5761 | 0.2336 | 0.1173 | 0.4037 | 0.0297 | 0.2384 | 0.5986 | 0.1298 | 0.9452 | 0.4996 |
| 26 | 0.1267 | 0.0447 | 0.0067 | 0.0985 | 0.0008 | 0.5590 | 0.7680 | 0.0437 | 0.5877 | 0.0008 |
| 27 | 0.1713 | -0.1590 | 0.1091 | -0.2382 | 0.1677 | 0.1611 | 0.5971 | 0.1901 | 0.9872 | 0.6824 |
| 28 | -0.3950 | 0.1512 | 0.2983 | 0.1794 | 0.3299 | 0.2931 | 0.7680 | 0.2255 | 0.8002 | 0.2571 |
| 29 | -0.3388 | -0.1727 | 0.0276 | -0.2266 | 0.0126 | -0.6384 | 0.2675 | 0.8207 | 0.1462 | 0.6156 |
| 30 | -0.3064 | 0.1363 | 0.1960 | 0.0469 | 0.1248 | -0.0902 | -0.2026 | 0.0126 | -0.0862 | 0.0000 |
| 31 | -0.0196 | -0.0421 | 0.0005 | -0.0792 | 0.0036 | -0.2065 | 0.2675 | 0.2247 | -0.1757 | 0.0009 |
| 32 | 0.1006 | 0.1658 | 0.0043 | 0.2115 | 0.0123 | -0.3002 | -0.0539 | 0.0607 | -0.1756 | 0.0155 |
| 33 | 0.1555 | -0.0630 | 0.0478 | -0.0667 | 0.0494 | -0.7370 | -0.1891 | 0.3002 | -0.2079 | 0.2799 |
| 34 | -0.2648 | 0.1002 | 0.1332 | 0.1944 | 0.2109 | -0.1375 | -0.1507 | 0.0002 | -0.2666 | 0.0167 |
| 35 | 0.1941 | 0.0203 | 0.0302 | 0.0593 | 0.0182 | -0.1279 | -0.1565 | 0.0008 | -0.1653 | 0.0014 |
| 36 | 0.0401 | 0.1143 | 0.0055 | 0.4022 | 0.1312 | -0.5590 | -0.1196 | 0.1930 | -0.4663 | 0.0086 |
| 37 | -0.4893 | 0.0808 | 0.3250 | -0.2047 | 0.081 | -0.2895 | -0.1799 | 0.0120 | -0.0981 | 0.0366 |
| 38 | 0.2125 | 0.1439 | 0.0047 | 0.3613 | 0.0221 | -0.0444 | -0.2042 | 0.0256 | -0.2468 | 0.0410 |
| **Mean** | **-0.0577** | **0.0532** | **0.0804** | **0.0529** | **0.0594** | **-0.1892** | **0.1138** | **0.2983** | **0.0126** | **0.2177** |

**Table S3** The mean square errors of the geo-accumulation index by the sequential Gaussian simulation

|  | The geo-accumulation index of As | | | The geo-accumulation index of Cd | | |
| --- | --- | --- | --- | --- | --- | --- |
| Sample NO. | Measured value | Sequential  Gaussian simulation | MSE | Measured value | Sequential  Gaussian simulation | MSE |
| 1 | 0.4857 | 0.3040 | 0.0330 | -0.1184 | -0.6579 | 0.2911 |
| 2 | 0.4517 | 0.6516 | 0.0400 | 0.2630 | 0.0418 | 0.0489 |
| 3 | 0.6664 | 0.2172 | 0.2018 | -0.2065 | -0.1503 | 0.0032 |
| 4 | 0.2135 | -0.0605 | 0.0751 | -0.9913 | 0.1286 | 1.2542 |
| 5 | 0.2771 | 0.4064 | 0.0167 | -0.7370 | -0.2848 | 0.2045 |
| 6 | 0.1460 | -0.4763 | 0.3872 | -0.0265 | 0.1403 | 0.0278 |
| 7 | -0.3934 | -0.3690 | 0.0006 | -0.0995 | -0.3806 | 0.0790 |
| 8 | -0.1546 | -0.1900 | 0.0013 | -0.0176 | 0.2035 | 0.0489 |
| 9 | -0.4860 | 0.1718 | 0.4327 | -0.4356 | 0.3415 | 0.6039 |
| 10 | -0.1559 | -0.0362 | 0.0143 | -0.2578 | -0.0567 | 0.0404 |
| 11 | 0.0100 | 0.1599 | 0.0225 | -0.3663 | -0.2512 | 0.0132 |
| 12 | -0.2747 | 0.0752 | 0.1225 | -0.1864 | -0.4286 | 0.0587 |
| 13 | -0.0570 | 0.0584 | 0.0133 | 0.7839 | -0.0820 | 0.7498 |
| 14 | -0.2890 | -0.1070 | 0.0331 | 0.1572 | -0.3000 | 0.2090 |
| 15 | -0.0595 | -0.5019 | 0.1957 | -0.2065 | -0.3153 | 0.0118 |
| 16 | -0.1428 | 0.0053 | 0.0219 | -0.1964 | 0.3793 | 0.3314 |
| 17 | 0.0297 | 0.5030 | 0.2240 | -0.4475 | -0.1623 | 0.0813 |
| 18 | -0.4152 | -0.0707 | 0.1187 | -0.3551 | 0.2537 | 0.3706 |
| 19 | -0.2977 | -0.0896 | 0.0433 | 0.1175 | 0.2398 | 0.0150 |
| 20 | -0.1823 | -0.6657 | 0.2336 | 0.1651 | 0.4209 | 0.0654 |
| 21 | -0.0376 | 0.5723 | 0.3719 | -0.2578 | 0.6538 | 0.8310 |
| 22 | -1.0136 | -0.1559 | 0.7357 | -0.3551 | 0.5839 | 0.8817 |
| 23 | -0.0339 | 0.1373 | 0.0293 | -0.6939 | 0.0880 | 0.6114 |
| 24 | -0.2410 | 0.1776 | 0.1752 | -0.8428 | 0.9807 | 3.3252 |
| 25 | 0.5761 | 0.0170 | 0.3126 | 0.2384 | 0.4162 | 0.0316 |
| 26 | 0.1267 | -0.2544 | 0.1452 | 0.5590 | 0.0237 | 0.2865 |
| 27 | 0.1713 | 0.0717 | 0.0099 | 0.1611 | 0.4087 | 0.0613 |
| 28 | -0.3950 | -0.2544 | 0.0198 | 0.2931 | 0.9075 | 0.3775 |
| 29 | -0.3388 | -0.5970 | 0.0667 | -0.6384 | -0.6794 | 0.0017 |
| 30 | -0.3064 | -0.1880 | 0.0140 | -0.0902 | -0.0302 | 0.0036 |
| 31 | -0.0196 | 0.1152 | 0.0182 | -0.2065 | -0.2582 | 0.0027 |
| 32 | 0.1006 | -0.6256 | 0.5274 | -0.3002 | -0.1972 | 0.0106 |
| 33 | 0.1555 | -0.3457 | 0.2512 | -0.7370 | -0.0413 | 0.4840 |
| 34 | -0.2648 | -0.0323 | 0.0541 | -0.1375 | 0.1136 | 0.0631 |
| 35 | 0.1941 | 0.2873 | 0.0087 | -0.1279 | 0.4883 | 0.3797 |
| 36 | 0.0401 | -0.3505 | 0.1525 | -0.5590 | -0.3260 | 0.0543 |
| 37 | -0.4893 | 0.1223 | 0.3741 | -0.2895 | -0.2028 | 0.0075 |
| 38 | 0.2125 | 0.5792 | 0.1345 | -0.0444 | -0.6886 | 0.4150 |
| **Mean** | **-0.0577** | **-0.0194** | **0.1482** | **-0.1892** | **0.0347** | **0.3246** |
